# Germline FOXJ2 overexpression causes male infertility via aberrant autophagy activation by LAMP2A upregulation

**DOI:** 10.1038/s41419-022-05116-w

**Published:** 2022-07-30

**Authors:** Fu-Rong Bai, Qi-Qian Wu, Yu-Jie Wu, Yan-Qin Hu, Zhi-Xuan Jiang, Hao Lv, Wen-Zhe Qian, Chang Cai, Jing-Wen Wu

**Affiliations:** 1 Department of Histoembryology, Genetics and Developmental Biology, Shanghai Jiao Tong University School of Medicine, 200025 Shanghai, China; 2 Shanghai Key Laboratory of Reproductive Medicine, 200025 Shanghai, China; 3 School of Medicine, Shanghai Jiao Tong University, 200025 Shanghai, China

**Keywords:** Spermatogenesis, Infertility

## Abstract

Spermatogenesis is a complex biological process that produces haploid spermatozoa and requires precise regulation by many tissue-specific factors. In this study, we explored the role and mechanism of Fork head box J2 (FOXJ2, which is highly expressed in spermatocytes) in the regulation of spermatogenesis using a germline-specific conditional *Foxj2* knock-in mouse model (*Stra8-Cre; Foxj2* ^*tg/tg*^ mouse). *Foxj2* overexpression in mouse testes led to spermatogenesis failure, which started at the initiation of meiosis, and resulted in male infertility. Lysosomes and autophagy-related genes were upregulated in *Stra8-cre; Foxj2* ^*tg/tg*^ mouse testes and the number of autolysosomes in the spermatocytes in *Stra8-cre; Foxj2* ^*tg/tg*^ mice was increased. Chromatin immunoprecipitation-PCR and Dual-luciferase reporter assays showed that *Lamp2* (encoding lysosome‐associated membrane protein‐2) was a target of FOXJ2. *Foxj2* overexpression increased the expression levels of *Lamp2a* and *Hsc70* (70-kDa cytoplasmic heat shock protein) in the *Stra8-cre; Foxj2* ^*tg/tg*^ mouse testes. Our results suggested that *Foxj2* overexpression in the germ cells of mouse testes affects chaperone-mediated autophagy by upregulating LAMP2A, leading to spermatogenesis failure at the initiation of meiosis, thus resulting in male infertility. Our findings provide a new insight into the function of FOXJ2 in spermatogenesis and the significance of autophagy regulation in spermatogenesis.

## Introduction

Spermatogenesis is a highly complex process comprising cell proliferation, differentiation, and migration, which is necessary to produce haploid spermatozoa. There are three main stages of spermatogenesis: mitosis of spermatogonia, meiosis of spermatocytes, and spermiogenesis of spermatids. Abnormalities in any stage may lead to failure of the entire spermatogenesis process, resulting in a reduced number or abnormal morphology of sperm, which will ultimately affect the reproduction and development of the individual. The successful progression of spermatogenesis is strictly controlled by accurate spatial and temporal regulation of gene expression, including transcriptional regulation through transcription factors binding to gene regulatory elements [1, 2].

Fork head box J2 (FOXJ2) is a transcription factor discovered in mammals and other vertebrates in 2000, which belongs to the Fork head box (Fox) transcription factor family, which shares a conserved DNA-binding domain, known as Fork Head [3, 4]. FOXJ2 participates in the regulation of cell proliferation, differentiation, and migration by targeting downstream genes through its DNA-binding domain, and plays fundamental roles in embryonic development, tumorigenesis, and the progression of certain cancers [5–7]. Previous studies demonstrated that *Foxj2* mRNA is specifically expressed in spermatocytes and round spermatids in mouse testis, but not in spermatogonia or elongating/elongated spermatids [8]. *Foxj2* overexpression (*Foxj2* transgenic mice) had a lethal effect on embryonic development. Among the small number of transgenic mice that survived to adulthood, only two mice (one male and one female) showed mosaic *Foxj2* expression and died at 9 w and 12 w, respectively, because of cardio-respiratory failure. The male transgenic mouse did not produce any offspring, while the female transgenic mouse had two pregnancies recorded. Histological analysis of the testes of this male transgenic mouse showed no mature spermatozoa in the seminiferous tubules, suggesting the failure of spermatogenesis. The mosaic and randomly integrated expression of the transgene, as well as the limitation of the number of samples (1 case) and observation time (9 w), made the results unconvincing [7]. Interestingly, our previous study and other research found that *Foxj2* mRNA was upregulated in the spermatocytes and round spermatids in miR-34b/c^−/−^; miR-449^−/−^ sterile mice [9, 10]. Furthermore, it was reported that loss of *Foxj2* in the male germline led to meiotic arrest and infertility via an as-yet-unknown mechanism [11].

The above research indicated that FOXJ2 participates in the regulation of spermatogenesis. However, which stage of spermatogenesis is affected by FOXJ2 and the regulation mechanism remain elusive. In the present study, using a germline-specific conditional *Foxj2* knock-in mouse model (*Stra8-cre; Foxj2* ^*tg/tg*^ mouse), we showed that *Foxj2* overexpression in the germ cells of mouse testes may affect chaperone-mediated autophagy (CMA) by upregulating lysosome-associated membrane protein 2A (LAMP2A), leading to failure of spermatogenesis starting at the initiation of meiosis, ultimately resulting in male infertility. Our data provide a new insight into the role of FOXJ2 in spermatogenesis and the significance of autophagy regulation in spermatogenesis.

## Results

### FOXJ2 is highly expressed in spermatocytes

**Fig. 1:**
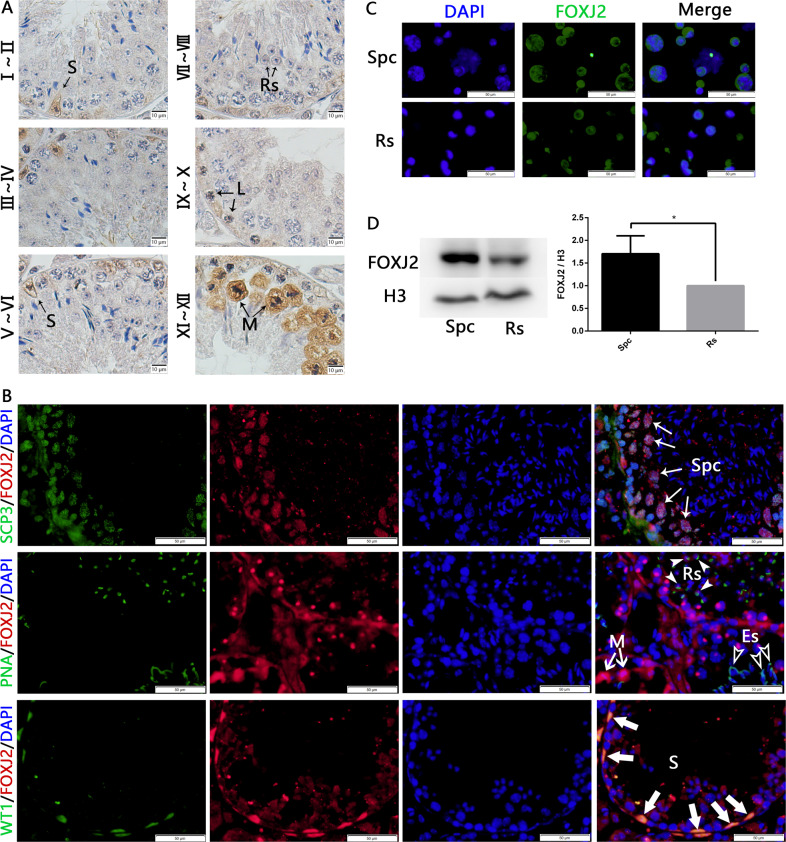
FOXJ2 localized to spermatocytes, round spermatids, and Sertoli cells in the seminiferous tubules and was highly expressed in spermatocytes. **A** Expression and localization of FOXJ2 in mouse testes using immunohistochemical staining (IHC). The roman numbers present the stages of the seminiferous epithelia. S Sertoli cell, Rs round spermatid, L leptotene spermatocyte, M metaphase of spermatocyte. Scale bar: 10 μm. **B** Immunofluorescent staining of SCP3 (showing spermatocytes) and FOXJ2, PNA (showing acrosomes) and FOXJ2, WT1(showing Sertoli cells) and FOXJ2 on testis sections of 8-week-old WT mice. Nuclei were counterstained with DAPI. Long white arrows indicate spermatocytes (Spc). White solid arrowheads indicate round spermatids (Rs). White hollow arrowheads indicate elongating spermatids (Es). Short white arrows indicate meiotic metaphase spermatocytes (M). White thick arrows indicate Sertoli cells (S). Scale bar: 50 μm. **C** Expression and localization of FOXJ2 in isolated spermatocytes (Spc) and round spermatids (Rs) using immunofluorescent staining (IF). **D** Western blotting analysis of FOXJ2 protein levels in isolated spermatocytes (Spc) and round spermatids (Rs) with the corresponding average gray levels. H3 was used as a loading control. Data are presented as the mean ± SD (*n* = 3). *P < 0.05.

We found that FOXJ2 was localized to spermatocytes, round spermatids, and Sertoli cells in the seminiferous tubules using immunohistochemical and immunofluorescent staining (Fig. 1A, B). To investigate the cellular localization of FOXJ2 in germ cells, we separated spermatogenic cells using STA-PUT velocity sedimentation [12]. Immunofluorescent staining for FOXJ2 revealed its expression in the nuclei of spermatocytes and round spermatids (Fig. 1C). Moreover, western blotting (WB) showed that FOXJ2 was highly expressed in spermatocytes, suggesting a critical role in meiosis (Fig. 1D).

### Overexpression of *Foxj2* in mouse testes leads to failure of spermatogenesis

**Fig. 2:**
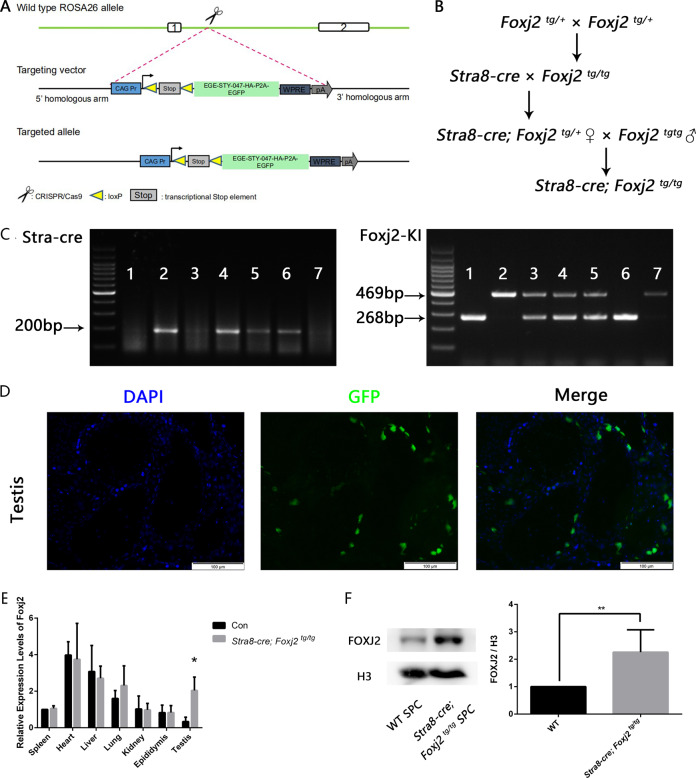
Conditional knock-in of *Foxj2* by Cre-loxP recombination. **A** Schematic depiction of gene targeting strategy for *Foxj2* knock-in (KI), among which “047” represents “*Foxj2* DNA”. A ROSA26-EGE-STY-047 cKI targeting vector was inserted behind the Lox-stop-Lox sequence. When crossed with the germline-specific Stra8-Cre line, the loxP sites will be recognized and excised by Cre, deleting the stop sequence, leading to overexpression of *Foxj2*. **B** Breeding strategy for *Foxj2*-cKI mice. **C** Genotypes of the mice: 1: *Foxj2* ^*tg/tg*^; 2: *Stra8-cre*; 3: *Foxj2* ^*tg/+*^; 4, 5: *Stra8-cre; Foxj2* ^*tg/+*^; 6: *Stra8-cre; Foxj2* ^*tg/tg*^; 7: wild-type (WT) mice. **D** The expression of GFP in the testes in *Stra8-cre; Foxj2* ^*tg/tg*^ mice. Scale: 100 μm. **E** Relative mRNA expression levels of *Foxj2* in different mouse tissues between *Stra8-cre; Foxj2* ^*tg/tg*^ and the control mice quantified by qRT-PCR. Data are presented as the mean ± SD (*n* = 3), normalized to *Actb* (β-actin) transcript levels. *P < 0.05. **F** Western blotting analysis of FOXJ2 protein levels in the isolated spermatocytes between *Stra8-cre; Foxj2* ^*tg/tg*^ and WT mice with analysis of gray values. H3 was used as a loading control. Data are presented as the mean ± SD (*n* = 3). **P < 0.01.

To explore the role of FOXJ2 in spermatogenesis, we generated a germline-specific conditional *Foxj2* knock-in mouse model (*Foxj2*-cKI) using the Cre-loxP recombination system (Fig. 2A, B). The successful generation of *Foxj2*-cKI mice was determined using PCR genotyping (Fig. 2C). In addition, the *Foxj2* overexpression vector carried a haemagglutinin (HA)-tag, which was fused with the target protein and expressed without affecting the biological activity and location of the target protein. In addition, the *Foxj2* overexpression vector expressed the green fluorescent protein (GFP), therefore the detection of GFP could reflect the overexpression of *Foxj2*. As expected, GFP was only detected in the seminiferous tubules in the testes (Fig. 2D), but not in other tissues of the *Stra8-cre; Foxj2* ^*tg/tg*^ mice (Supplementary Fig. S1A). Quantitative real-time reverse transcription PCR (qRT-PCR) demonstrated the testis-specific overexpression of *Foxj2*, whose expression levels did not change significantly in other tissues (Fig. 2E). WB analysis showed higher levels of FOXJ2 in the spermatocytes of *Stra8-cre; Foxj2* ^*tg/tg*^ mice than in the wild-type (WT), confirming the tissue-specific overexpression of FOXJ2 (Fig. 2F).

**Fig. 3:**
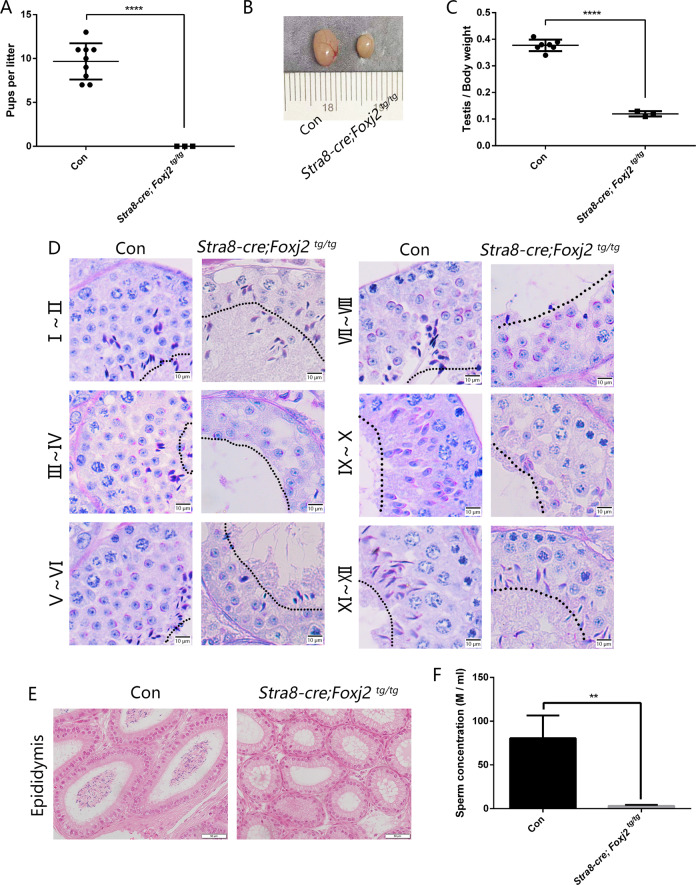
*Stra8-cre; Foxj2*^*tg/tg*^ mice were sterile due to reduced numbers of meiotic and post-meiotic cells in the testis and almost no sperm in the epididymis. **A** Pups per litter sired by Con and *Stra8-cre; Foxj2* ^*tg/tg*^ males. ****P < 0.0001. **B** Gross morphology of the testes from Con and *Stra8-cre; Foxj2* ^*tg/tg*^ males. **C** Ratio of the testis to body weight from Con and *Stra8-cre; Foxj2* ^*tg/tg*^ males. ****P < 0.0001. **D** Micrographs of PAS-stained testes sections from Con and *Stra8-cre; Foxj2* ^*tg/tg*^ mice. The roman numbers present the stages of the seminiferous epithelia. Dashed lines indicate the apical borders of the seminiferous epithelia. Scale bar: 10 μm. **E** Micrographs of H&E-stained epididymides sections from Con and *Stra8-cre; Foxj2* ^*tg/tg*^ mice. Scale bar: 50 μm. **F** Comparison of sperm concentration in the cauda epididymis between the Con and *Stra8-cre; Foxj2* ^*tg/tg*^ by CASA. Data are presented as the mean ± SD (*n* = 3). **P < 0.01.

To test whether *Foxj2* overexpression has an impact on male fertility, 2-month-old adult *Stra8-cre; Foxj2* ^*tg/tg*^ males, as well as the control male littermates, were mated with fertility-proven adult females. The result showed that the control male littermates could produce an average of 9.67 ± 2.06 pups per litter, whereas *Stra8-cre; Foxj2* ^*tg/tg*^ males failed to produce a pregnancy and were completely sterile (Fig. 3A), which suggested that overexpression of *Foxj2* has a negative effect on male fertility. The testis size and relative weight (testis weight/body weight) of the *Stra8-cre; Foxj2* ^*tg/tg*^ mice decreased significantly compared with that of the control mice (Fig. 3B, C), which was likely caused by the loss of germ cells. Indeed, Periodic acid-Schiff (PAS) staining on testes sections showed that the numbers of spermatocytes and spermatids were significantly reduced in the seminiferous tubules of *Stra8-cre; Foxj2* ^*tg/tg*^ mice (Fig. 3D), resulting in almost no sperm in the epididymis (Fig. 3E) and a decreased sperm concentration in the *Stra8-cre; Foxj2* ^*tg/tg*^ mouse epididymis (Fig. 3F). Together, these results account for the infertility phenotype observed in the *Stra8-cre; Foxj2* ^*tg/tg*^ males.

**Fig. 4:**
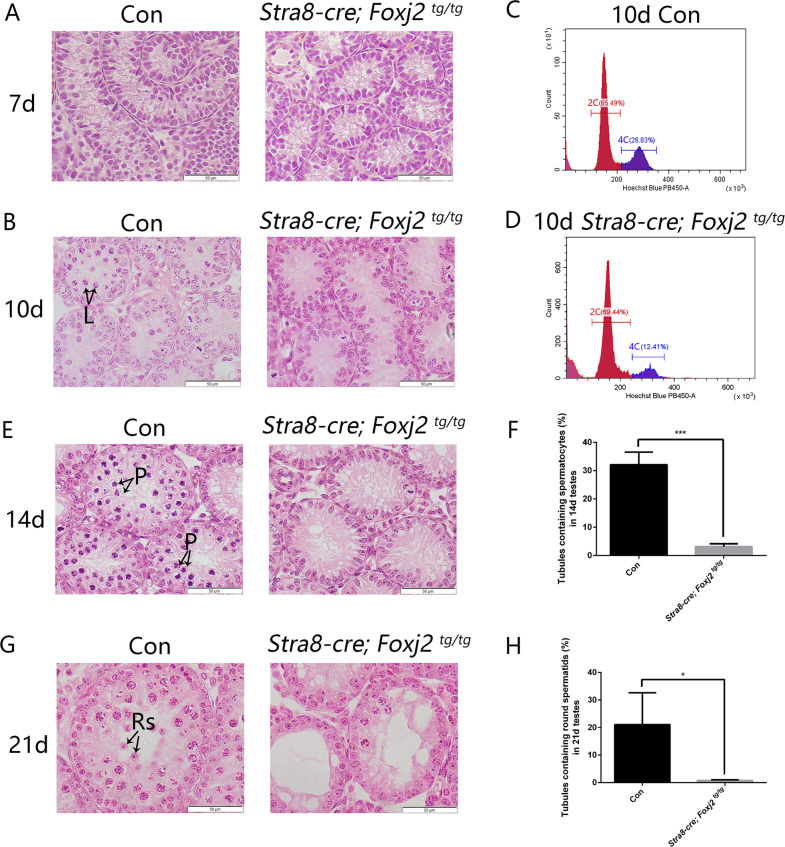
Morphology of the developing testes revealed histological differences beginning from postnatal day 10. **A**, **B**, **E**, **G** Micrographs of H&E-stained testicular sections from Con and *Stra8-cre; Foxj2* ^*tg/tg*^ mice at postnatal day 7 (7 d) (**A**), 10 d (**B**), 14 d (**E**), and 21 d (**G**). L-leptotene spermatocytes; P-pachytene spermatocytes; Rs-round spermatids; Scale bar: 50 μm. **C** Flow cytometry analysis of testicular cell suspensions from 10-day-old (10 d) Con mice. Red-2C DNA content (65.49%), Blue-4C DNA content (26.83%). **D** Flow cytometry analysis of testicular cell suspensions from 10-day-old (10 d) *Stra8-cre; Foxj2* ^*tg/tg*^ mice. Red-2C DNA content (69.44%), Blue-4C DNA content (12.41%). **F** The percentage of tubules containing pachytene spermatocytes in the seminiferous tubules from testicular sections between 14-day-old Con and *Stra8-cre; Foxj2* ^*tg/tg*^ mice. Data are presented as the mean ± SD (*n* = 3). ***P < 0.001. **H** The percentage of tubules containing round spermatids in the seminiferous tubules from testicular sections between 21-day-old Con and *Stra8-cre; Foxj2* ^*tg/tg*^ mice. Data are presented as the mean ± SD (*n* = 3). *P < 0.05.

There are only spermatogonia and Sertoli cells in the seminiferous epithelium of postnatal day 6 mice. Meiosis occurs on day 10, with the appearance of preleptotene spermatocytes (pL) and leptotene spermatocytes (L), reaching the pachytene stage by day 14. Haploid spermatids appear around day 21 [13–15]. To determine which step of spermatogenesis is first impacted by *Foxj2* overexpression, we observed developing testes in the prepubertal mice. By 7 days after birth, there were only spermatogonia and Sertoli cells in the testes of both *Stra8-cre; Foxj2* ^*tg/tg*^ mice and the control littermates (Fig. 4A). When germ cells enter meiosis on day 10, morphological differences began to appear between the *Stra8-cre; Foxj2* ^*tg/tg*^ mice and their control littermates (Fig. 4B). Quantitative analysis of the testicular cells in 10-day-old mice using flow cytometry showed that the spermatocytes (4C cells) accounted for about 12.41% of the total number of testicular cells in the *Stra8-cre; Foxj2* ^*tg/tg*^ mice, which was about half of that in the control mice (26.83%) (Fig. 4C, D), which confirmed the decrease of primary spermatocytes in the 10-day-old *Stra8-cre; Foxj2* ^*tg/tg*^ mice. On day 14, early pachytene spermatocytes appeared in some (32.09% ± 4.45%) seminiferous tubules in the testes of the control mice, while only a few (3.16% ± 1.02%) seminiferous tubules contained pachytene spermatocytes in the *Stra8-cre; Foxj2* ^*tg/tg*^ mice (Fig. 4E, F). By day 21, round spermatids were present in some (21.09% ± 11.57%) seminiferous tubules of the control mice, while there were almost no round spermatids (0.81% ± 0.24%) in the *Stra8-cre; Foxj2* ^*tg/tg*^ testes, but many vacuoles were observed (Fig. 4G, H). Observation of the developing testes showed that the histological differences of the testes between the *Stra8-cre; Foxj2* ^*tg/tg*^ mice and their control littermates began from postnatal day 10 when the spermatogenic cells enter meiosis, suggesting that overexpression of *Foxj2* in germ cells led to a failure of meiosis initiation during spermatogenesis.

### FOXJ2 affects spermatocytes' autophagy by targeting *Lamp2*

To explore the molecular mechanism of spermatogenic failure in the *Stra8-cre; Foxj2* ^*tg/tg*^ mice, we conducted RNA-sequencing (RNA-seq) to analyze the transcriptome alterations in the germ cells of *Stra8-cre; Foxj2* ^*tg/tg*^ mice. Given that there were few spermatocytes and spermatids in the seminiferous tubules of adult *Stra8-cre; Foxj2* ^*tg/tg*^ mouse testes, and the morphological difference between the *Stra8-cre; Foxj2* ^*tg/tg*^ and control mice began from postanal day 10, we performed RNA-seq on the testicular cells of the 10-day-old *Stra8-cre; Foxj2* ^*tg/tg*^ and WT mice.

**Fig. 5:**
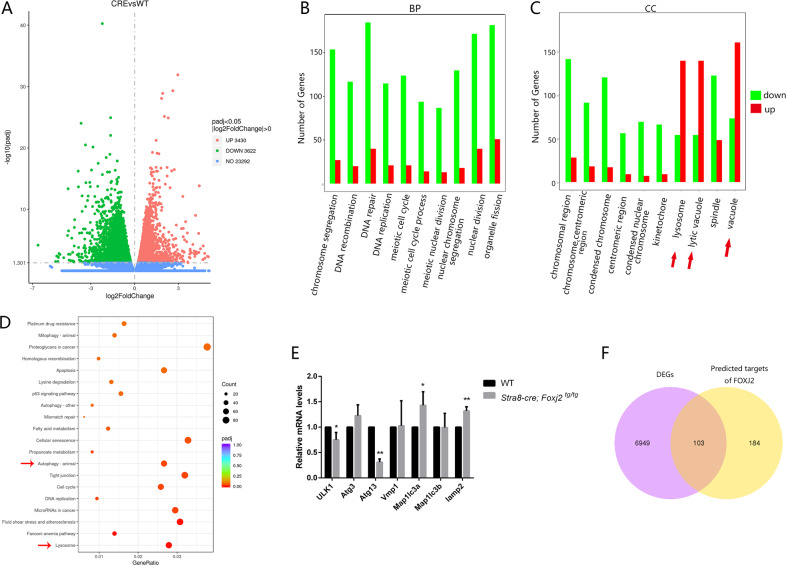
Analysis of the transcriptome alteration in the testes of *Stra8-cre; Foxj2*^*tg/tg*^ mice. **A** Volcano diagram of differentially expressed genes. Red: 3430 upregulated genes; Green: 3622 downregulated genes; Blue: 23292 genes with no significant differences in expression. **B**–**D** Functional enrichment analysis of DEGs, including GO category biological process (**B**), cellular component (**C**), and KEGG pathway (**D**). Red arrows point to genes with significant differences. **E** Validation of DEGs related to the autophagy pathway by qRT-PCR. Data are presented as the mean ± SD (*n* = 3). *P < 0.05; **P < 0.01. **F** Venn diagram of DEGs (purple) and the predicted target genes of FOXJ2 (yellow).

The RNA-seq data presented 7052 differentially expressed genes (DEGs) in the *Stra8-cre; Foxj2* ^*tg/tg*^ mice as compared with those in the WT, including 3430 upregulated genes and 3622 downregulated genes, which was in line with the role of FOXJ2 as a transcriptional regulator (Fig. 5A). The Gene Ontology (GO) analysis of the DEGs showed that the genes participating in the Biological Processes (BP) involved in meiosis, such as DNA replication, DNA repair, and DNA recombination, were significantly downregulated (Fig. 5B), which was consistent with the infertility phenotype of the *Stra8-cre; Foxj2* ^*tg/tg*^ males with reduced spermatocytes in the seminiferous tubules. Notably, in the Cellular Component (CC) category, most of the genes related to lysosomes and vacuoles were upregulated in the *Stra8-cre; Foxj2* ^*tg/tg*^ mouse testes (Fig. 5C). Meanwhile, the Kyoto Encyclopedia of Genes and Genomes (KEGG) analyses of these DEGs showed that expression levels of genes involved in the lysosome and autophagy pathways were significantly different between the *Stra8-cre; Foxj2* ^*tg/tg*^ and WT mice (Fig. 5D). We then detected the expression levels of genes involved in autophagy pathways using qRT-PCR. The results showed that the mRNA expression levels of *Ulk1* (encoding Unc-51-like kinase 1) and *Atg13* (autophagy-related gene 13), which are related to autophagy initiation, were decreased, while the mRNA expression levels of *Map1lc3a* (microtubule-associated protein 1 light chain 3 alpha), related to autophagosome extension, and *Lamp2*, related to autophagosome maturation, were upregulated significantly in *Stra8-cre; Foxj2* ^*tg/tg*^ mouse testes (Fig. 5E) [16–19]. Furthermore, to screen the potential target genes regulated by FOXJ2 in the testes, we obtained 287 predicted target genes of FOXJ2 through bioinformatic analyses using Transcription Factor Binding Site (TFBS) Tools (1.18.0) software [20]. Only 103 out of 287 predicted target genes were identified among the DEGs dysregulated between the *Stra8-cre; Foxj2* ^*tg/tg*^ and WT mouse testes (Fig. 5F) and GO analyses of the differentially expressed target genes revealed the genes related to lysosomes and lytic vacuoles had significantly different expression levels (Supplementary Fig. S2A).

**Fig. 6:**
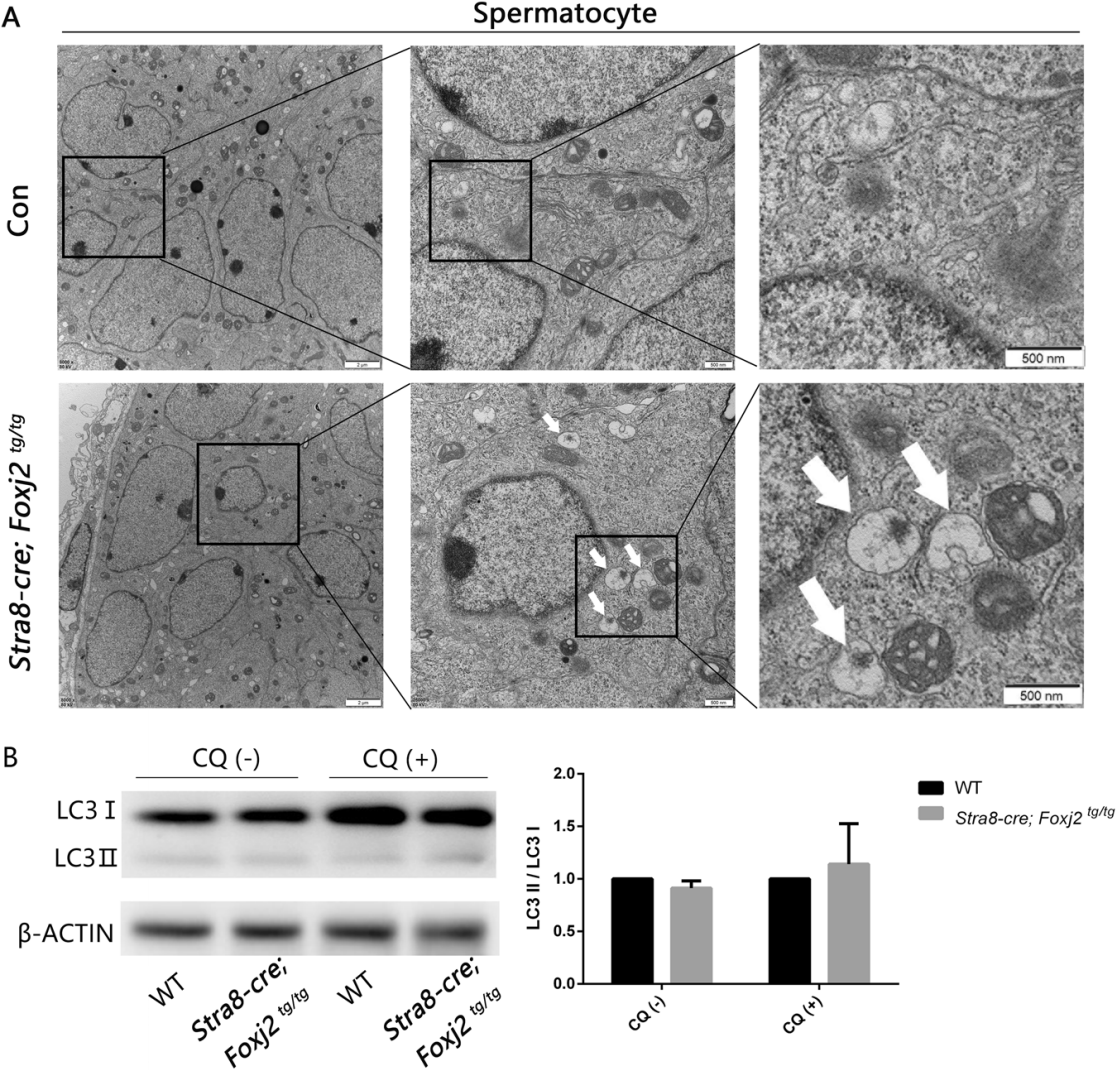
Autolysosomes increased in the spermatocytes in 10-day-old *Stra8-cre; Foxj2*^*tg/tg*^ mouse testes. **A** Transmission electron microscopy images of spermatocytes from 10-day-old *Stra8-cre; Foxj2* ^*tg/tg*^ and control mice. White arrows indicate autolysosomes. Scale bar: 2 μm (left), 500 nm (middle and right). **B** Western blotting analysis of LC3 II/I protein levels with or without CQ (chloroquine) treatment in wild-type (WT) and *Stra8-cre; Foxj2* ^*tg/tg*^ mouse testes followed by gray values analysis. β-Actin was used as a loading control. Data are presented as the mean ± SD (*n* = 3).

As mentioned above, among the DEGs, the genes related to lysosomes and autophagy were upregulated significantly in *Stra8-cre; Foxj2* ^*tg/tg*^ mouse testes, as compared to the WT. We speculated that the autophagy in the *Stra8-cre; Foxj2* ^*tg/tg*^ mouse testes was dysregulated. Therefore, we observed the ultrastructure of the testes from 10-day-old *Stra8-cre; Foxj2* ^*tg/tg*^ mice and their control littermates using a transmission electron microscope, and observed more autolysosomes in the spermatocytes in the *Stra8-cre; Foxj2* ^*tg/tg*^ mice than in their control littermates (Fig. 6A), while there was no difference in the number of autolysosomes in the spermatogonia and Sertoli cells between the *Stra8-cre; Foxj2* ^*tg/tg*^ mice and their control littermates (Supplementary Fig. S3A, B). To detect whether autophagosome formation was different between the *Stra8-cre; Foxj2* ^*tg/tg*^ and control mice, we detected the level of microtubule-associated protein 1 light chain 3 alpha isoform II (LC3 II) using WB [21], and found that the levels of LC3 II/LC3 I did not change. To rule out the impact of LC3 II degradation after autophagosomes fuse with lysosomes in vivo, we injected chloroquine (CQ, an inhibitor of lysosomes) into the mice one day in advance [22], and did not find any change in LC3 II levels (Fig. 6B). The above results suggested that the level of autophagy in the spermatocytes of *Stra8-cre; Foxj2* ^*tg/tg*^ mice was increased, but there was no obstacle to the formation of autophagosomes, which is a critical process during the macroautophagy.

**Fig. 7:**
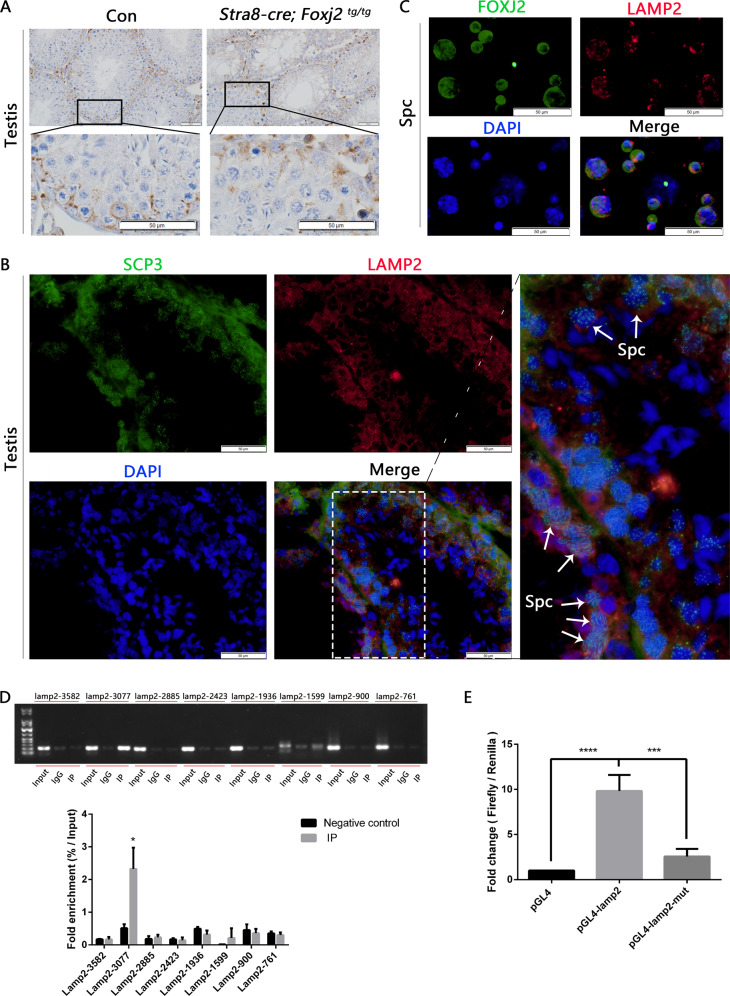
LAMP2 co-localized with FOXJ2 in spermatocytes and was a target of FOXJ2. **A** Expression and localization of LAMP2 in testis using immunohistochemical staining (IHC). Scale bar: 50 μm. **B** Localization of LAMP2 and SCP3 (showing spermatocytes) on testis sections using immunofluorescent staining. Scale bar: 50 μm. White arrows indicate spermatocytes (Spc). **C** Localization of LAMP2 and FOXJ2 in isolated spermatocytes using immunofluorescent staining (IF). Scale bar: 50 μm. **D** The binding sites of FOXJ2 to *Lamp2* promoter region in the testicular cells of the 10-day-old *Stra8-cre; Foxj2* ^*tg/tg*^ mice analyzed by ChIP-PCR using anti-HA-tag (ChIP level). Data are presented as the mean ± SD (*n* = 3). *P < 0.05. **E** Verification of FOXJ2 binding to the *Lamp2* promoter using a Dual-luciferase reporter assay. Data are presented as the mean ± SD (*n* = 3). ***P < 0.001; ****P < 0.0001.

We next sought to explore the potential target genes regulated by FOXJ2 in the testis. As shown above, there were more autolysosomes in the spermatocytes in the *Stra8-cre; Foxj2* ^*tg/tg*^ mice (Fig. 6A). The validation of gene expression changes for autophagy-related DEGs from the RNA-seq data showed that the expression of *Lamp2* mRNA was increased significantly in the *Stra8-cre; Foxj2* ^*tg/tg*^ mouse testes (Fig. 5E). In addition, *Lamp2* was predicted to be the target gene of FOXJ2 by bioinformatic analyses (Fig. 5F). Taken together, these data suggested a mechanistic link between FOXJ2 and *Lamp2* in spermatocyte autophagy, which warranted further investigation. First, we observed the location of LAMP2 in the testes using immunohistochemical and immunofluorescent staining and found that LAMP2 was localized to spermatocytes and Sertoli cells (Fig. 7A, B and Supplementary Fig. S4A). Whether LAMP2 and FOXJ2 were co-located in spermatocytes was further detected using the isolated spermatocytes (Fig. 7C). The results demonstrated that both LAMP2 and FOXJ2 were located in spermatocytes.

To further confirm whether FOXJ2 affected spermatogenesis by targeting *Lamp2*, we examined the binding of FOXJ2 to the promoter of *Lamp2* in vivo using chromatin immunoprecipitation-PCR (ChIP-PCR). Considering that there is no ChIP-level anti-FOXJ2 antibody on the market, and the *Foxj2* overexpression vector carried an HA-tag (Fig. 2A), we used a ChIP-level anti-HA-tag antibody to conduct ChIP-PCR. The RNA-seq results were based on the testicular cells from 10-day-old *Stra8-cre; Foxj2* ^*tg/tg*^ and WT mice; therefore, we chose to use the testicular cell suspension from 10-day-old *Stra8-cre; Foxj2* ^*tg/tg*^ mice as the experimental sample for ChIP-PCR. The results showed that in the testicular cells of the 10-day-old *Stra8-cre; Foxj2* ^*tg/tg*^ mice, the fold enrichment of FOXJ2 binding to its binding site in the *Lamp2* promoter region amplified by the *Lamp2-3077* primers (later termed the Lamp2-3077 site) was significantly higher than that of the negative control (Fig. 7D), which suggested that FOXJ2 can bind to the *Lamp2-3077* site. Therefore, we choose the *Lamp2-3077* site for further verification using a dual-luciferase reporter assay in vitro.

HeLa cells endogenously express FOXJ2 (Supplementary Fig. S4B); therefore, there was no need to add a FOXJ2 expression plasmid when performing the dual-luciferase reporter assay. After transfecting the pGL4-Lamp2 plasmid containing the *Lamp2-3077* binding site into HeLa cells for 24 h, the luciferase activity was significantly higher than that of the control group (pGL4-basic). When the *Lamp2-3077*-binding site was mutated, the luciferase activity was reduced significantly (Fig. 7E). These results indicated that FOXJ2 directly bound to the *Lamp2-3077* site in the promoter region of the *Lamp2* gene to upregulate its expression.

**Fig. 8:**
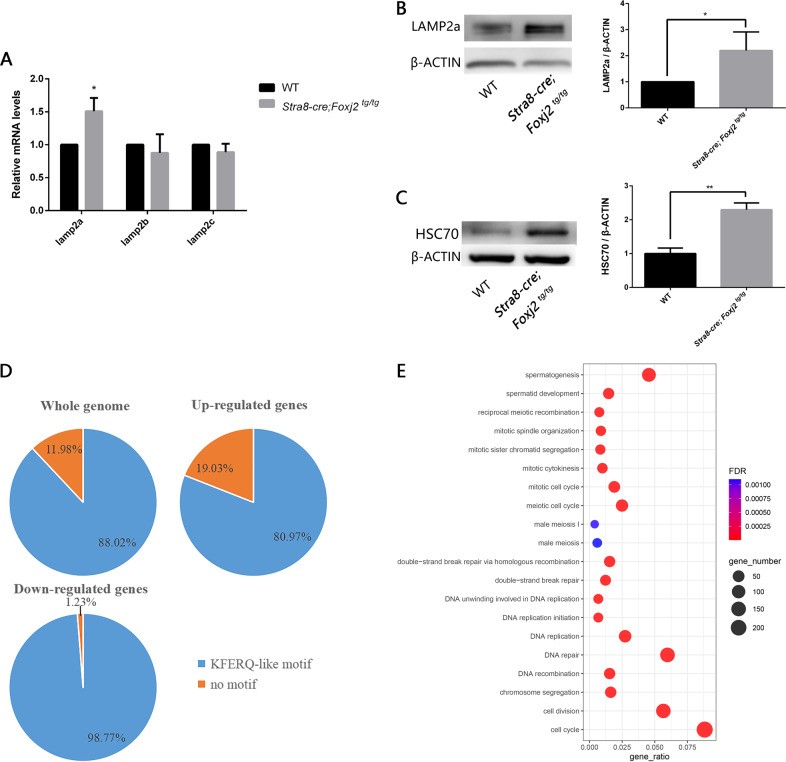
CMA pathway involved in the failure of spermatogenesis in the *Stra8-cre; Foxj2*^*tg/tg*^ mouse testes. **A** Relative mRNA expression levels of *Lamp2a*, *Lamp2b*, *Lamp2c* in 10-day-old wild-type (WT) and *Stra8-cre; Foxj2* ^*tg/tg*^ mouse testes quantified by qRT-PCR. Data are presented as the mean ± SD (*n* = 3). *P < 0.05. **B**, **C** Western blotting analysis of LAMP2A (**B**) and HSC70 (**C**) protein levels in 10-day-old WT and *Stra8-cre; Foxj2* ^*tg/tg*^ mice testes with analysis of gray values. β-Actin was used as a loading control. Data are presented as the mean ± SD (*n* = 3). *P < 0.05; **P < 0.01. **D** The pro*p*ortion of the proteins containing KFERQ-like motifs encoded by the whole genome genes, upregulated genes, and downregulated genes based on our RNA-seq data. **E** GO analysis of the downregulated genes that encode the proteins containing KFERQ-like motifs.

As mentioned above, the number of autolysosomes in the spermatocytes of *Stra8-cre; Foxj2* ^*tg/tg*^ mice was increased, but there was no obstacle to the formation of autophagosomes. We then asked the question: what is the effect of overexpression of *Foxj2* on the autophagy process at the initiation of meiosis in spermatogenesis? According to the literature, there are three different isoforms of LAMP2 arising from alternative mRNA splicing, known as LAMP2A, LAMP2B, and LAMP2C, which function in different types of autophagy, namely CMA, macroautophagy, and RNautophagy/DNautophagy, respectively [23, 24]. We then tested the expression of the isoforms of *Lamp2* in the testis and found that the mRNA expression level of *Lamp2a* in the testes of *Stra8-cre; Foxj2* ^*tg/tg*^ mice was significantly higher than that of the WT, while there were no significant differences in the mRNA expression levels of *Lamp2b* or *Lamp2c* between the *Stra8-cre; Foxj2* ^*tg/tg*^ and WT mice (Fig. 8A). WB verified the increased level of LAMP2A protein in the testes of *Stra8-cre; Foxj2* ^*tg/tg*^ mice (Fig. 8B). At the same time, the levels of the 70-kDa cytoplasmic heat shock protein (HSC70), the cytosolic chaperone required by CMA to form a complex with the substrate protein that recognized by LAMP2A to be delivered to the lysosome for degradation [25], also increased in the testes of the *Stra8-cre; Foxj2* ^*tg/tg*^ mice (Fig. 8C). CMA is a selective process that targets and degrades proteins containing a pentapeptide motif (KFERQ-like motif) [25]. To clarify whether CMA involves in the failure of spermatogenesis in the *Stra8-cre; Foxj2* ^*tg/tg*^ mice, we performed an in silico screen for KFERQ-like motifs based on our RNA-seq results using the KFERQ finder [26]. We found that the proportion of proteins containing KFERQ-like motifs encoded by the downregulated genes (98.77%) was higher than the proportion of proteins encoded by the upregulated genes (80.97%) or by whole genome genes (88.02%), implying that more proteins encoded by the downregulated genes were prone to be degraded by CMA in the *Stra8-cre; Foxj2* ^*tg/tg*^ mice (Fig. 8D). In addition, we performed a GO enrichment analysis in terms of the downregulated genes that encoded the proteins containing the KFERQ-like motifs. Notably, it revealed that these genes were mainly involved in several biological processes, including mitosis, meiosis, cell cycle, DNA repair/recombination, and spermatogenesis (Fig. 8E), furtherly supporting that CMA involved in the failure of spermatogenesis through degradation of the substrate proteins in the *Stra8-cre; Foxj2* ^*tg/tg*^ mice. The above results indicated that the increase in the number of autolysosomes in the spermatocytes of the *Stra8-cre; Foxj2* ^*tg/tg*^ mice might be caused by the effects of the abnormally increased expression of *Lamp2a* on the CMA pathway.

## Discussion

In this study, we showed that overexpression of *Foxj2* in the male germline led to male infertility as a result of the reduced meiotic and post-meiotic cells in the adult *Stra8-cre; Foxj2* ^*tg/tg*^ mouse testes and the abnormality began at postnatal day 10, indicating a failure of spermatogenesis, which started at the initiation of meiosis. The transcriptome alterations of the testicular cells between 10-day-old *Stra8-cre; Foxj2* ^*tg/tg*^ and WT mice showed that genes related to lysosomes and autophagy were significantly upregulated in *Stra8-cre; Foxj2* ^*tg/tg*^ mouse testes. At the same time, an increased number of autolysosomes in the spermatocytes of *Stra8-cre; Foxj2* ^*tg/tg*^ mice was observed, indicating aberrant autophagy activation in the spermatocytes of the *Stra8-cre; Foxj2* ^*tg/tg*^ mice.

Autophagy is a conserved, lysosome-dependent catabolic process with the primary function of degrading and recycling cytoplasmic components to maintain cellular homeostasis [27]. Three main types of autophagy have been identified according to the different ways by which the cytoplasmic components are delivered to lysosomes, namely macroautophagy, microautophagy, and CMA [28]. Macroautophagy involves the formation of the double membrane vesicles, called autophagosomes, which subsequently fuse with lysosomes, forming autolysosomes. It comprises four sequential stages, known as initiation, nucleation, maturation, and degradation [29]. Microautophagy is a non-selective degradation process that directly swallows intracellular components into lysosomes [27]. In CMA, the protein substrate containing the KFERQ-like pentapeptide sequence is recognized by a chaperone protein, HSC70, to form a complex, which binds to LAMP2A on the lysosome membrane and is then transferred to the lysosome [17, 25]. Interestingly, we found that *Lamp2*, a target of FOXJ2, which was verified by ChIP-PCR and Dual-luciferase reporter assays, was significantly upregulated in *Stra8-cre; Foxj2* ^*tg/tg*^ mouse testes.

LAMP2 is a major lysosomal membrane protein, which is an important regulator of the maturation of autophagosomes and phagosomes [19, 30]. There are three different isoforms of LAMP2 arising by alternative mRNA splicing, known as LAMP2A, LAMP2B, and LAMP2C, which have the same luminal domain, but different transmembrane regions and cytoplasmic tails [24]. LAMP2A is highly expressed in tissues such as the placenta, lung, liver, kidney, and pancreas, and is considered to be a receptor on the lysosomal membrane in the CMA process [31]. LAMP2B is more abundantly expressed in the heart, skeletal muscle, and brain [32]. LAMP2B might be related to macroautophagy and affect the maturation of autophagosomes [33]. LAMP2C is mainly expressed in the brain, eyes, heart, liver, kidney, and skeletal muscle. LAMP2C is a receptor for selective RNA and DNA degradation autophagy, namely RNautophagy and DNautophagy, respectively [34, 35]. Our results showed that the expression levels of LAMP2A and HSC70 were increased in the *Stra8-cre; Foxj2* ^*tg/tg*^ mouse testes. In addition, the bioinformatics analysis demonstrated that more proteins encoded by the downregulated genes contained the KFERQ-like motifs in the *Stra8-cre; Foxj2* ^*tg/tg*^ mice. These proteins containing KFERQ-like motifs involved in mitosis, meiosis, DNA repair/recombination, and spermatogenesis, implying that activation of CMA abnormally degraded the substrate proteins necessary for spermatogenesis in the *Stra8-cre; Foxj2* ^*tg/tg*^ mice. The above results suggested that overexpression of *Foxj2* can upregulate the expression of *Lamp2a*, causing aberrant CMA activation, which leads to the increased number of autolysosomes in the spermatocytes in *Stra8-cre; Foxj2* ^*tg/tg*^ mice. The mechanisms underlying the regulation of CMA in spermatogenesis, especially during the transition from mitosis to meiosis, are currently unclear. One possibility is that CMA suppression should be controlled at the initiation of meiosis during spermatogenesis, which warrants further investigation.

Increasing evidence shows that autophagy involved in spermatogenesis [36–39]. It was reported that germ cells lacking *Stra8* (encoding stimulated by retinoic acid 8) failed to enter meiosis, and showed autophagy activation. This revealed the connection between STRA8-mediated autophagy suppression and the initiation of meiosis [40]. Moreover, the level of autophagy increased in high-fat diet mice with disrupted spermatogenesis and male fertility, and autophagy was also overactivated in sperm samples from obese subfertile male patients [41, 42]. Recent studies have found that LAMP2 was highly expressed in human spermatogonia, and some autophagy-related genes were dysregulated in the testicular germ cells of patients with non-obstructive azoospermia (NOA), suggesting that autophagy is related to male infertility [43].

In summary, our data showed that overexpression of *Foxj2* in the germ cells of mouse testis might affect CMA by upregulating *Lamp2a*, leading to a failure of spermatogenesis, starting at the initiation of meiosis, and resulting in male infertility. Our findings provide a new insight into the function of FOXJ2 in spermatogenesis and the significance of autophagy regulation in spermatogenesis.

## Materials and methods

### Animals

Stra8-cre mice were purchased from the Nanjing Biomedical Research Institute of Nanjing University. *Foxj2* knock-in mice (*Foxj2* ^tg/tg^) were generated by crossing *Foxj2* ^tg/+^ mice, which were prepared by Beijing Biocytogen Co., Ltd. (Beijing, China). All lines were maintained in the C57BL/6J background. Genotypes of the mice were identified by PCR using DNA extracted from mice tails. The primers used to detect Stra8-Cre and *Foxj2* knock-in in this assay are listed in Table S1. All control (Con) mice in this study were littermates of the analyzed *Foxj2*-cKI mice. All animal experiments were performed with the approval of the Institutional Animal Care and Use Committee of the Shanghai Jiao Tong University School of Medicine.

### Histology, immunohistochemistry, and immunofluorescence staining

For hematoxylin and eosin (H&E) staining, freshly dissected testes and epididymides were fixed in Bouin’s solution overnight at room temperature. These tissues were embedded in paraffin after dehydration and cut into 5-μm sections, followed by H&E staining after deparaffinization and rehydration. Periodic Acid-Schiff (PAS) staining was performed according to the manufacturer’s instructions (Beyotime, Shanghai, China). For immunohistochemistry staining, the testes were fixed in 4% paraformaldehyde and paraffin-embedded. Then, 5-μm sections were cut and rehydrated, followed by antigen retrieval in 10 mM citrate buffer (pH 6.0) for 15 min. The sections were blocked in 5% bovine serum albumin (BSA) for 1 h at room temperature, after being treated with 3% H_2_O_2_, and incubated overnight at 4 °C with anti-FOXJ2 antibodies (1:200, ab22857, Abcam, Cambridge, MA, USA), or anti-Lamp2 antibodies (1:200, ab13524, Abcam). On the following day, the sections were incubated with goat anti-rabbit/rat IgG antibody for 1 h at room temperature, followed by diaminobenzidine and hematoxylin counterstaining. Images were observed under a microscope (Nikon, Tokyo, Japan). For immunofluorescence staining, 4% paraformaldehyde-fixed samples were embedded in optimal cutting temperature compound (OCT) and then cut in 8-μm cryo-sections. After blocking in 5% BSA for 1 h at room temperature, the sections were incubated with primary antibodies recognizing FOXJ2 (1:200, ab22857, Abcam), SCP3 (Synaptonemal complex protein 3) (1:200, ab97672, Abcam), WT1 (Wilms Tumor 1) (1:200, ab89901, Abcam) or Lamp2 (1:200, ab13524, Abcam) at 4 °C overnight, followed 1 h of incubation at room temperature with Alexa fluor 488/594-labeled secondary antibody or PNA (Lectin from Arachis hypogaea/Peanut lectin) (1:400, L7381, Sigma). After counterstaining with 4’, 6-diamidino-2-phenylindole (DAPI), these tissues were observed under a fluorescence microscope (Leica, Germany).

### Isolation of germ cells by STA-PUT

Testicular germ cells were isolated by STA-PUT according to the publication with minor modification [12]. Testes from adult mice were decapsulated and incubated in 10 ml Dulbecco’s modified Eagle’s medium (DMEM) (Gibco, Grand Island, NY, USA) containing 100 µl of collagenase IV (Sigma, St. Louis, MO, USA), 0.015 g of hyaluronidase (Sigma), 1 ml of trypsin (Sigma), and 100 µl of DNAse I (Sigma) under gentle agitation at 34 °C for about 45 min with pipetting at the mid-time point until the testes were digested into cell suspension. After centrifugation, the cells were resuspended in 25 ml 0.5% BSA solution and filtered through a 40-µm filter to obtain a single-cell suspension. Then, the cell suspension was loaded into the cell chamber, with 550 ml of 2% BSA and 550 ml of 4% BSA solution in the two cylinders, respectively. The stopcocks were opened to allow the 4% BSA, 2% BSA, and 0.5% BSA containing the cells to flow slowly into the sedimentation chamber. About 40 min after the solutions were loaded into the sedimentation chamber, the valve was turned off, followed by 3 h of sedimentation. Next, the cells in the sedimentation chamber were collected and examined under a microscope. Fractions containing cells with similar sizes and morphologies were combined for subsequent examination.

### Protein preparation and western blotting analysis

Samples were homogenized in radioimmunoprecipitation assay (RIPA) lysis buffer (Thermo Fisher Scientific, Waltham, MA, USA) containing protease inhibitor cocktail (Roche Applied Science, Basel, Switzerland) on ice for around 30 min for total protein extraction. The nucleoprotein was separated according to the manufacturer’s instructions, using a Nuclear and Cytoplasmic Protein Extraction Kit (Sangon Biotech, China). The proteins were collected in the supernatant after centrifugation at 14,000 × *g* for 10 min at 4 °C, and the protein concentrations were determined using a bicinchoninic acid (BCA) Protein Assay Kit (Thermo Fisher Scientific). The protein samples were separated using 10% sodium dodecyl sulfate–polyacrylamide gel electrophoresis (SDS-PAGE), and then transferred to polyvinylidene difluoride membranes (Millipore, Billerica, MA, USA). The membranes were blocked in 5% BSA for 1 h at room temperature, followed by incubation in primary antibodies recognizing FOXJ2 (1:500, ab22857, Abcam), LC3 (1:1000, Cell Signaling Technology, Danvers, MA, USA), LAMP2A (1:1000, ab125068, Abcam), H3 (Histon H3) (1:1000, ab1791, Abcam) and β-actin (1:1000, ab8227, Abcam), respectively, overnight at 4 °C. On the second day, the membranes were incubated with secondary antibodies conjugated to horseradish peroxidase (HRP) (1:5000, Cell Signaling Technology) for 1 h at room temperature. The signals were visualized via enhanced chemiluminescence (Millipore) and detected by a luminescent image analyzer (ImageQuant LAS 4000 mini, Chicago, IL, USA). The results were quantified using ImageJ (version 1.46r; National Institutes of Health, Bethesda, MD, USA) and normalized to the level of H3 or β-actin.

### RNA extraction and qRT-PCR

Total RNAs were extracted using an RNAsimple Total RNA Kit (TIANGEN, Beijing, China) according to the manufacturer’s instructions. For reverse transcription, cDNA was prepared from 1 μg of RNA using PrimeScript RT Master Mix (Takara, Dalian, China). The quantitative real-time PCR reaction was prepared using the TB Green Premix Ex Taq II (Takara) in accordance with the manufacturer’s protocol and performed using an Applied Biosystems 7500 instrument (ABI, Foster City, CA, USA). Relative quantification of the mRNA levels was calculated using the threshold cycle (CT) method 2^−∆∆Ct^ with *Actb* (encoding β-actin) as the endogenous control [44]. The primers sequences used in this study are listed in Supplementary Table S2, and were synthesized by Sangon Biotech (Shanghai, China).

### Fertility evaluation

Each adult *Stra8-cre; Foxj2* ^*tg/tg*^ male or their control male littermate was mated with two female mice with normal fertility according to the ratio of one male to two fertility-proven adult females for at least 1 year. The numbers of pups per litter and the date of delivery were recorded.

### Computer-assisted sperm analysis (CASA)

For each sample, the cauda epididymis was harvested from the adult mouse, and incubated in Human Tubel Fluid (HTF) (Millipore) at 37 °C for 15 min to release the sperm. The supernatant was collected for the evaluation of sperm counts using the CASA system (Hamilton Thorne, Beverly, MA, USA).

### Flow cytometry analysis

A testicular cell suspension from 10-day-old mice was prepared as described above. The cells were stained with 10 mg/ml Hoechst 33342 (Thermo Fisher) for 30 min and propidium iodide (PI) (Invitrogen, Waltham, MA, USA) for 5 min before examination using CytoFlex S (Beckman, Indianapolis, IN, USA). The results were analyzed using CytExpert 2.0 (Beckman).

### RNA-seq and bioinformatic analysis

Testes from 10-day-old mice were harvested for RNA extraction. The quality of the RNA samples was analyzed by detection of RNA purity (A_260_/A_280_) and integrity. Complementary DNA library construction and quality inspection were performed using the above mRNA as the template. According to the effective concentration and data requirements, the library was pooled and then sequenced using the Illumina system (Illumina, San Diego, CA, USA). Then, DEG analysis and functional enrichment analysis, including GO and KEGG pathway analysis, were carried out. The target genes of FOXJ2 were predicted by using TFBS Tools (1.18.0) software [20].

### Transmission electron microscopy analysis

Testes from 10-day-old mice were immersed in 2.5% glutaraldehyde in 0.1 M phosphate buffer (pH 7.4) overnight at 4 °C and postfixed in 1% osmium tetroxide for 2 h at 4 °C. After dehydration, the samples were embedded in Epon 618 (TAAB Laboratories Equipment, Aldermaston, UK) and cut to ultrathin sections (70–90 nm), followed by staining with uranyl acetate and lead citrate. The ultrastructure of germ cells in the testes was observed using a transmission electron microscope (Philips CM-120, Amsterdam, the Netherlands).

### ChIP-PCR

ChIP was performed using a ChIP Assay kit (Millipore) according to the manufacturer’s instructions. In brief, after preparation of single-cell suspensions of testes using DMEM containing collagenase IV, hyaluronidase, and DNase I, the cells were cross-linked in 1% formaldehyde for 10 min at room temperature and then neutralized using glycine. The cells were then lysed on ice in the lysis buffer with proteinase inhibitors, and the nuclear lysates were sonicated to break the genome into 200–1000 bp fragments. The lysate was immunoprecipitated with primary antibodies (anti-HA-tag) or control IgG at 4 °C for 1 h, and then captured by protein A/G beads at 4 °C overnight, followed by washing with different washing buffers. The DNA components in the precipitation were finally extracted using a standard phenol-chloroform method. Purified chromatin-immunoprecipitated DNA was subjected to PCR analysis using primers to amplify the FOXJ2 binding sites in the *Lamp2* promoter region, which are listed in Supplementary Table S3.

### Dual-luciferase reporter assay

For FOXJ2 target gene validation, the pGL4-Lamp2 plasmid was constructed for the *Lamp2-3077* site “CTGTTTA” to which FOXJ2 binds, as shown in the ChIP-PCR results (Fig. 7E). The pGL4-Lamp2-Mutant plasmid containing the mutated *Lamp2-3077* binding site “GCCCACC” was generated using a QuickChange Lightning Site-Directed Mutagenesis Kit (Agilent Technologies, Santa Clara, CA, USA). HeLa cells were cultured in 12-well plates and were co-transfected with pGL4-Lamp2 or pGL4-Lamp2-Mutant plasmids using Lipofectamine 3000 reagent (Invitrogen). Renilla luciferase was transfected for normalization of the transfection efficiency. After 24 h of transfection, the Dual-Luciferase Reporter Assay System (Promega, Madison, WI, USA) was used to measure the activities of firefly luciferase and Renilla luciferase using the cell lysates, according to the manufacturer’s protocol. The activity of firefly luciferase was normalized to that of Renilla luciferase.

### Identification of KFERQ-like motifs

To identify the KFERQ-like motifs in the substrate proteins encoded by the differentially expressed genes of the RNA-seq data, the gene IDs were firstly transformed to the Uniprot ID using the Uniprot (www.uniprot.org). The KFERQ-like motifs were then retrieved from the KFERQ finder V0.8 [26]. GO enrichment analysis was performed based on the downregulated genes which encode proteins containing the KFERQ-like motifs.

### Statistical analysis

The data are presented as the mean ± SD and were analyzed using GraphPad Prism 6 (GraphPad Inc., La Jolla, CA, USA). Group comparisons were analyzed using Student’s *t* test where appropriate. *P* < 0.05 was considered statistically significant. All experiments were performed in triplicate.

## Supplementary information

aj-checklist

Original Data File

Supplemental Data

## Data Availability

The RNA-seq data have been deposited into the National Center for Biotechnology Information Sequence Read Archive database (accession no. PRJNA824792).
