# Supplementary material for: Germline FOXJ2 overexpression causes male infertility via aberrant autophagy activation by LAMP2A upregulation
Source: Cell Death Dis. 2022 Jul 30;13(7):665. doi: 10.1038/s41419-022-05116-w (PMC9338950; doi:10.1038/s41419-022-05116-w)
Supplement: Supplementary file 2 — Original Data File [file 41419_2022_5116_MOESM2_ESM.docx]

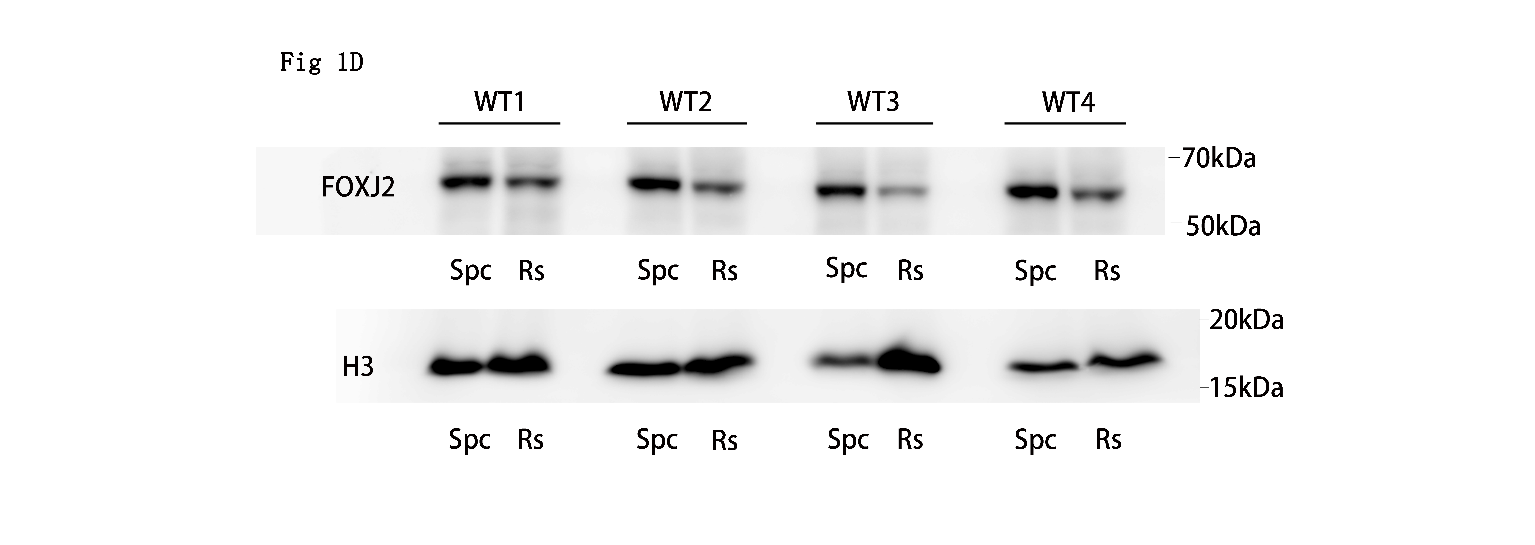


**Fig.1D** Western blotting analysis of FOXJ2 protein levels in isolated spermatocytes (Spc) and round spermatids (Rs). H3 was used as a loading control.


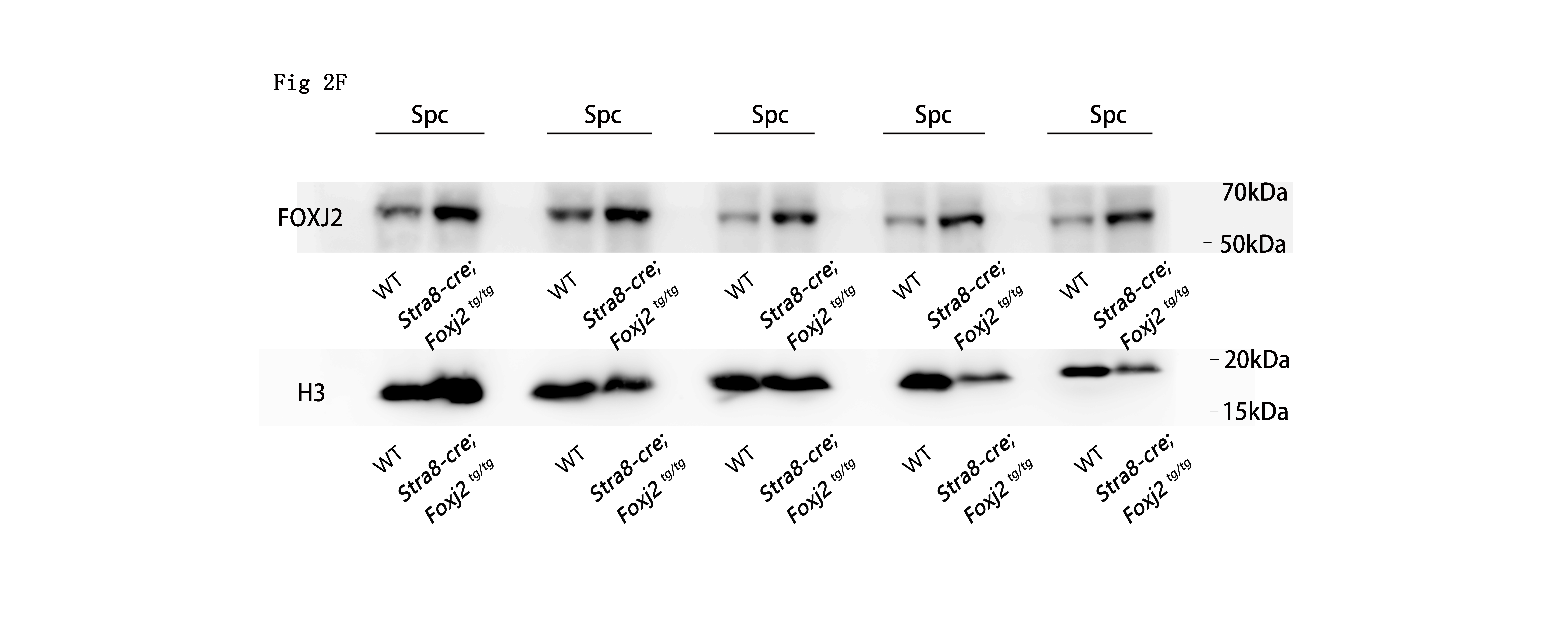


**Fig. 2F** Western blotting analysis of FOXJ2 protein levels in the isolated spermatocytes between *Stra8-cre; Foxj2 ^tg/tg^* and WT mice. H3 was used as a loading control.


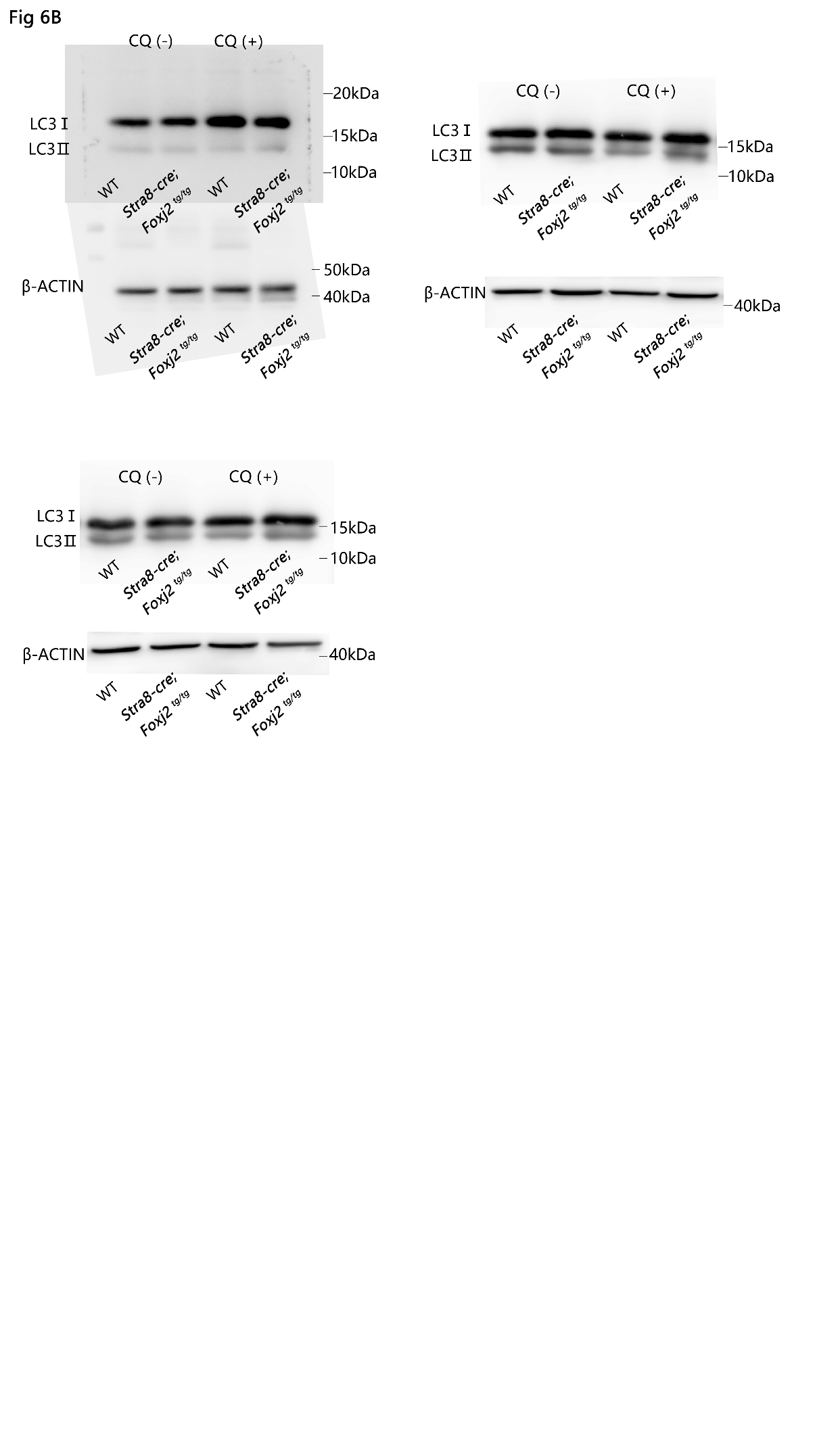


**Fig. 6B** Western blotting analysis of LC3Ⅱ/Ⅰ protein levels with or without CQ (chloroquine) treatment in wild-type (WT) and *Stra8-cre; Foxj2 ^tg/tg^* mouse testes. β-Actin was used as a loading control.


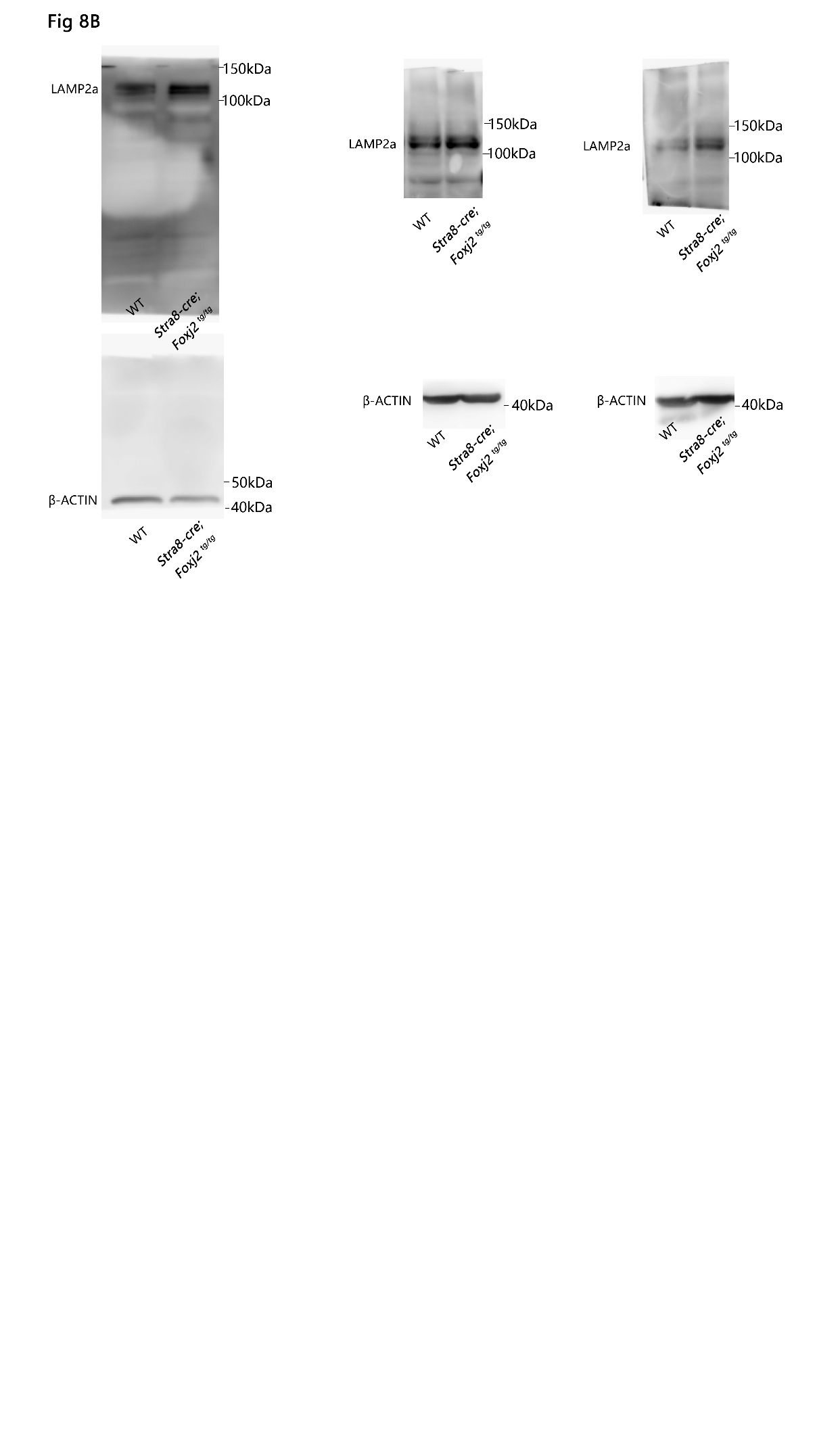


**Fig. 8B** Western blotting analysis of LAMP2A protein levels in 10-day-old WT and *Stra8-cre; Foxj2 ^tg/tg^* mice testes. β-Actin was used as a loading control.


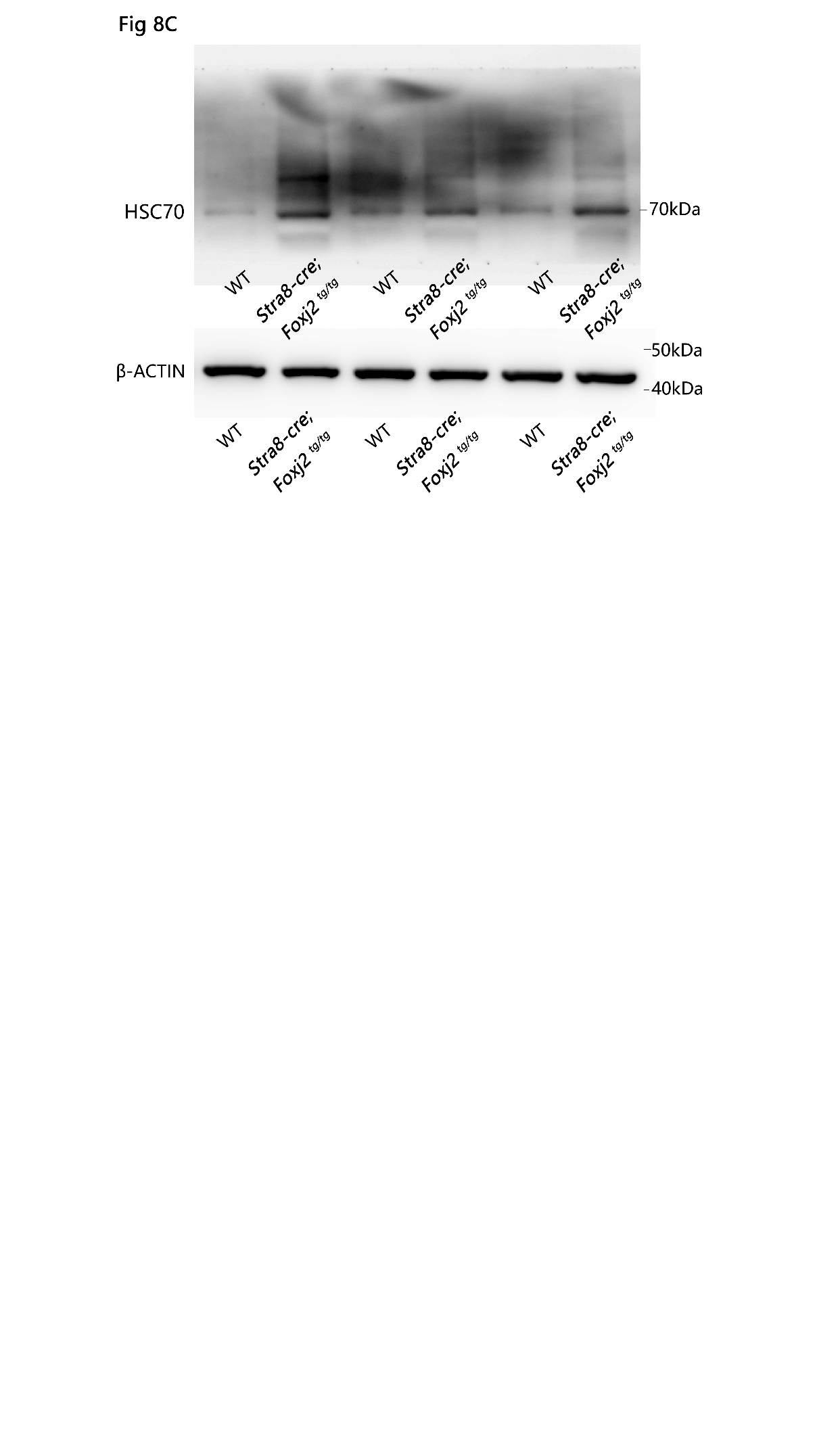


**Fig. 8C** Western blotting analysis of HSC70 protein levels in 10-day-old WT and *Stra8-cre; Foxj2 ^tg/tg^* mice testes. β-Actin was used as a loading control.
