# Supplementary material for: Germline FOXJ2 overexpression causes male infertility via aberrant autophagy activation by LAMP2A upregulation
Source: Cell Death Dis. 2022 Jul 30;13(7):665. doi: 10.1038/s41419-022-05116-w (PMC9338950; doi:10.1038/s41419-022-05116-w)
Supplement: Supplementary file 3 — Supplemental Data [file 41419_2022_5116_MOESM3_ESM.docx]

**Supplemental Data**

Table S1. Primer sequences for Genotyping

| Name | Primer sequence | |
| --- | --- | --- |
| Stra8 | F: 5’- GTGCAAGCTGAACAACAGGA -3’ | R: 5’- AGGGACACAGCATTGGAGTC -3’ |
| ROSA-GT | F: 5’- AGTCGCTCTGAGTTGTTATCAG -3’ | R: 5’- TGAGCATGTCTTTAATCTACCTCGATG -3’ |
| ROSA-PCR |  | R: 5’- AGTCCCTATTGGCGTTACTATGG -3’ |

Table S2. Primer sequences for qRT-PCR

| Name | Primer sequence | |
| --- | --- | --- |
| *Foxj2* | F: 5’-GATACAACCGCCCAGCACA-3’ | R: 5’- GTCCCAGTCGAAGTCATCGG-3’ |
| *Lamp2* | F: 5’- CTTAGCTTCTGGGATGCCCC -3’ | R: 5’- GCACTGCAGTCTTGAGCTGT -3’ |
| *Map1lc3a* | F: 5’- CAGCTTCGCCGACCG-3’ | R: 5’- TGGTCTGGGACCAGAAACTTG -3’ |
| *Map1lc3b* | F: 5’- AGGGGACCCTAACCCCATAG -3’ | R: 5’- CGCTCTATAATCACCCGCCT-3’ |
| *Vmp1* | F: 5’- AGAAGTGGGGAGGAAACCAGA -3’ | R: 5’- CATGAACTGAAGAGGGGTCTGT -3’ |
| *Atg13* | F: 5’- GACCCCTACTCCTGTGGTGA -3’ | R: 5’- GCGAGAGCTTGAGAGTTGATG -3’ |
| *Atg3* | F: 5’- GAAGTGGCCGAGTACCTGAC-3’ | R: 5’- CTTCCCCTGTAGCCCTCTTCT-3’ |
| *ULK1* | F: 5’- AAAACCTGCAATCGCCCACT-3’ | R: 5’- TGGTAGGAAAAGGGTGGCTTG -3’ |
| *Lamp1* | F: 5’- GCCCTGGAATTGCAGTTTGG -3’ | R: 5’- TGCTGAATGTGGGCACTAGG -3’ |
| *Actb* | F: 5’- ACTGTCGAGTCGCGTCCA-3’ | R: 5’- ATCCATGGCGAACTGGTGG-3’ |

Table S3. Specific primers for amplifying the FOXJ2 binding sites in the promoter region of *Lamp2*

| Name | Primer sequence | |
| --- | --- | --- |
| Lamp2-2066 | F: 5’- CCCCTTTGGTAAACTCTATCTCC -3’ | R: 5’- GGCCTTGACTTTGGTGATGT -3’ |
| Lamp2-3077 | F: 5’- CCTCCATACGCACACAGGT -3’ | R: 5’- ACTAGGCTTGTTTTAGGTTGGT -3’ |
| Lamp2-2885 | F: 5’- CAAGCCTAGTTACTGTGATGGT -3’ | R: 5’- TTCTTCTTCAGGGAGCTGCA -3’ |
| Lamp2-2423 | F: 5’- GCAGGAATGGTAGGATGTGC -3’ | R: 5’- ACCTCAGAACACCACATTTTCT -3’ |
| Lamp2-1936 | F: 5’- CCTCACAGAGATCCTCCTGC -3’ | R: 5’- CAAAGTGTGCCCTACGAATGT -3’ |
| Lamp2-1599 | F: 5’- TCTGTGAGTTCTAGGCCAGC -3’ | R: 5’- CCATGAGACACTGGTGAGGA -3’ |
| Lamp2-900 | F: 5’- GGCAACTTAGTGAGGATTAAACC -3’ | R: 5’- ACACACACACACACACACAC -3’ |
| Lamp2-761 | F: 5’- GTGTGTGTGTGTGTGTGTGT -3’ | R: 5’- ATTCTGTACGCTTGCCAAGT -3’ |


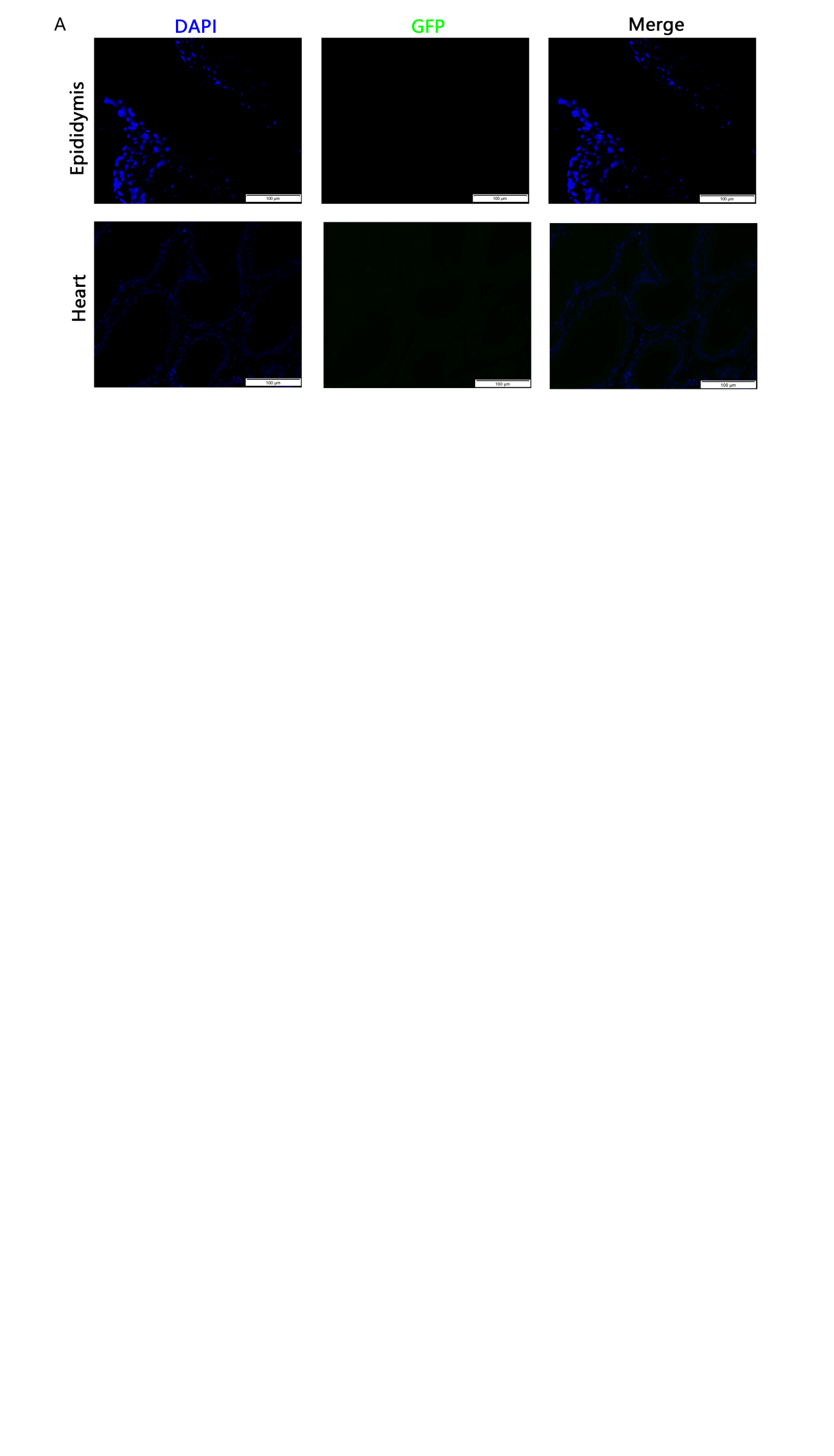


**Supplementary Fig. S1. GFP was not detected in the epididymis or the heart of the *Stra8-cre; Foxj2 ^tg/tg^* mice.** (A) The expression of GFP in different tissues in the *Stra8-cre; Foxj2 ^tg/tg^* mice. Scale: 100 μm.


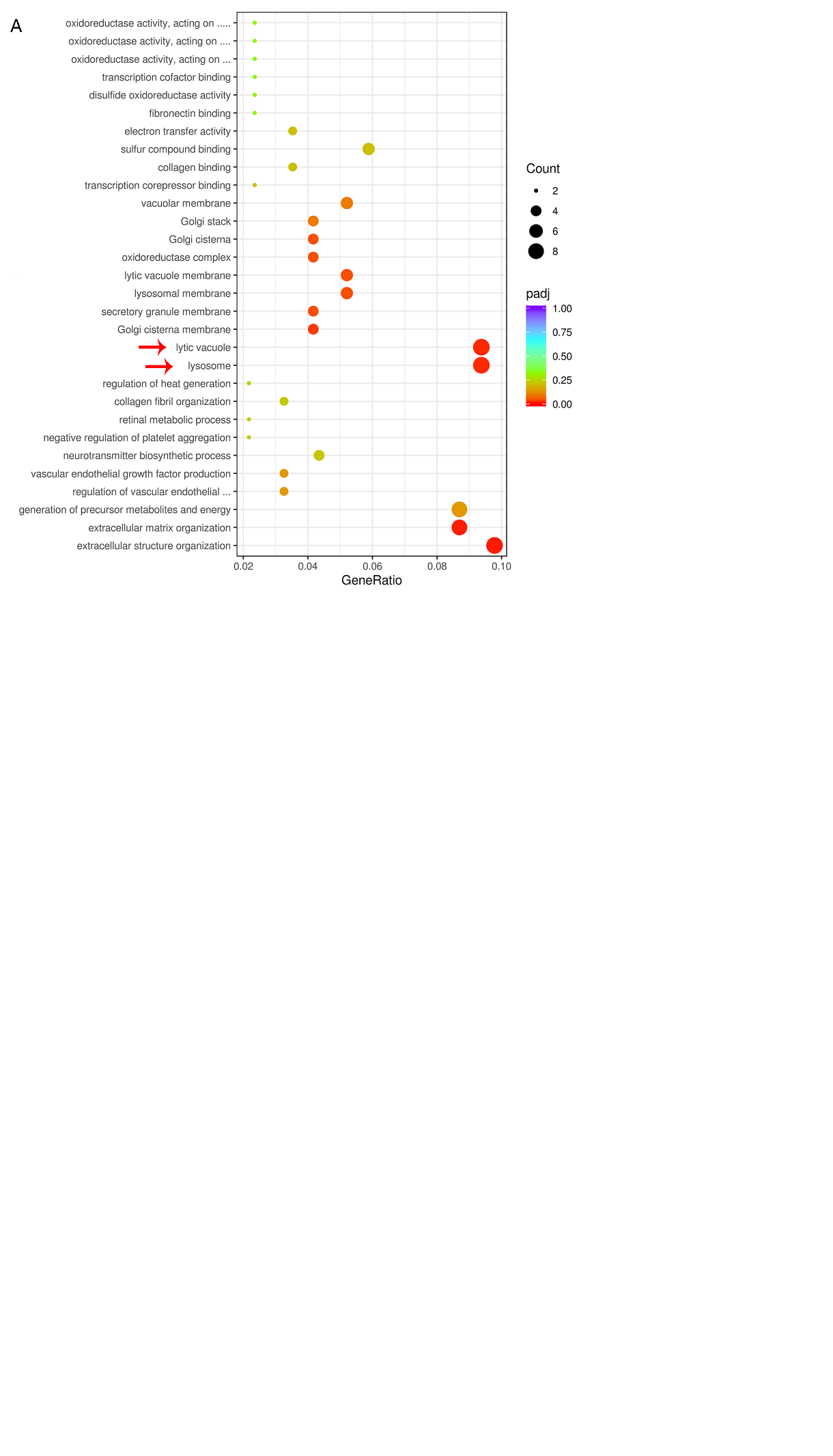


**Supplementary Fig. S2. The differentially expressed target genes related to lysosomes and lytic vacuoles had significant differences.** (A) GO analyses of the differentially expressed target genes. Red arrows point to genes with significant differences.


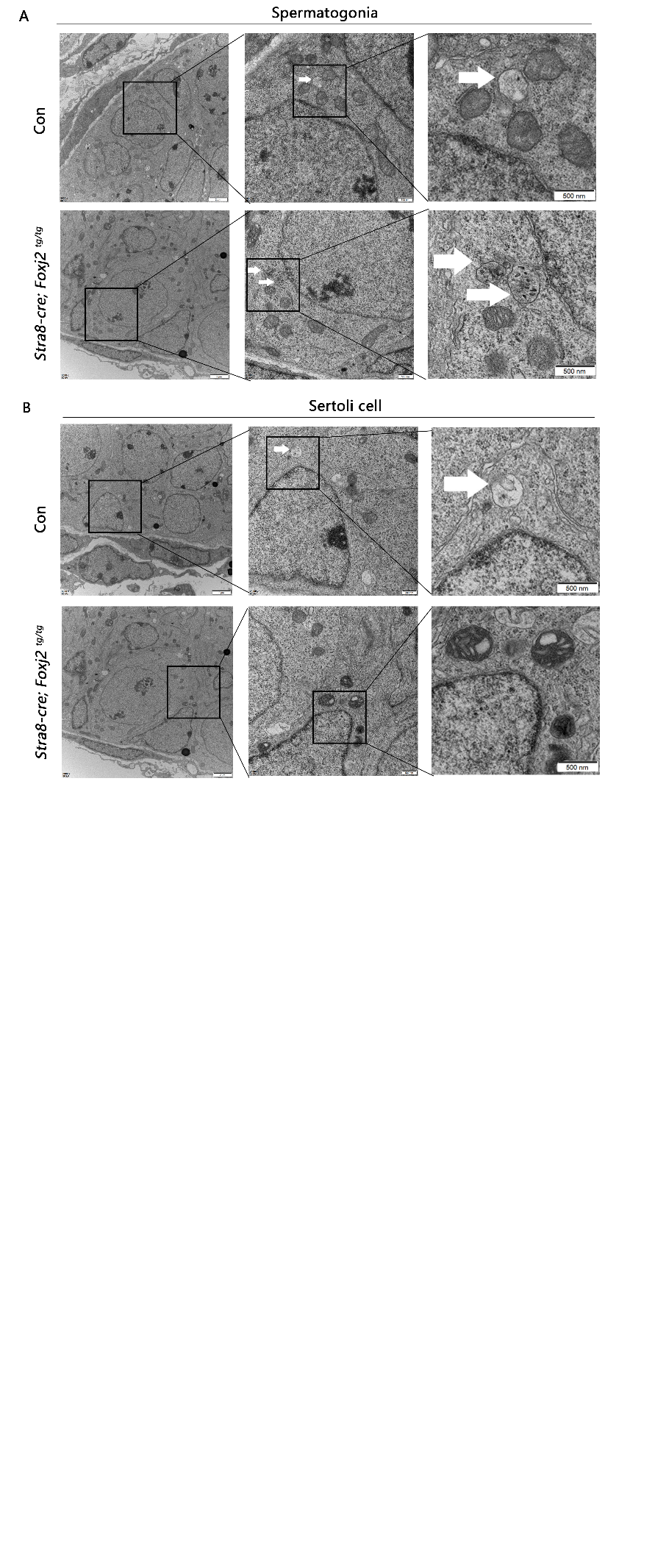


**Supplementary Fig. S3. There was no difference in the number of autolysosomes in the spermatogonia and Sertoli cells between the *Stra8-cre; Foxj2 ^tg/tg^* mice and their control littermates.** (A-B) Transmission electron microscope images of spermatogonia (A) and Sertoli cells (B) from 10-day-old *Stra8-cre; Foxj2 ^tg/tg^* mice and control mice. White arrows indicate autolysosomes. Scale bar: 2 μm (left), 500 nm (middle and right).


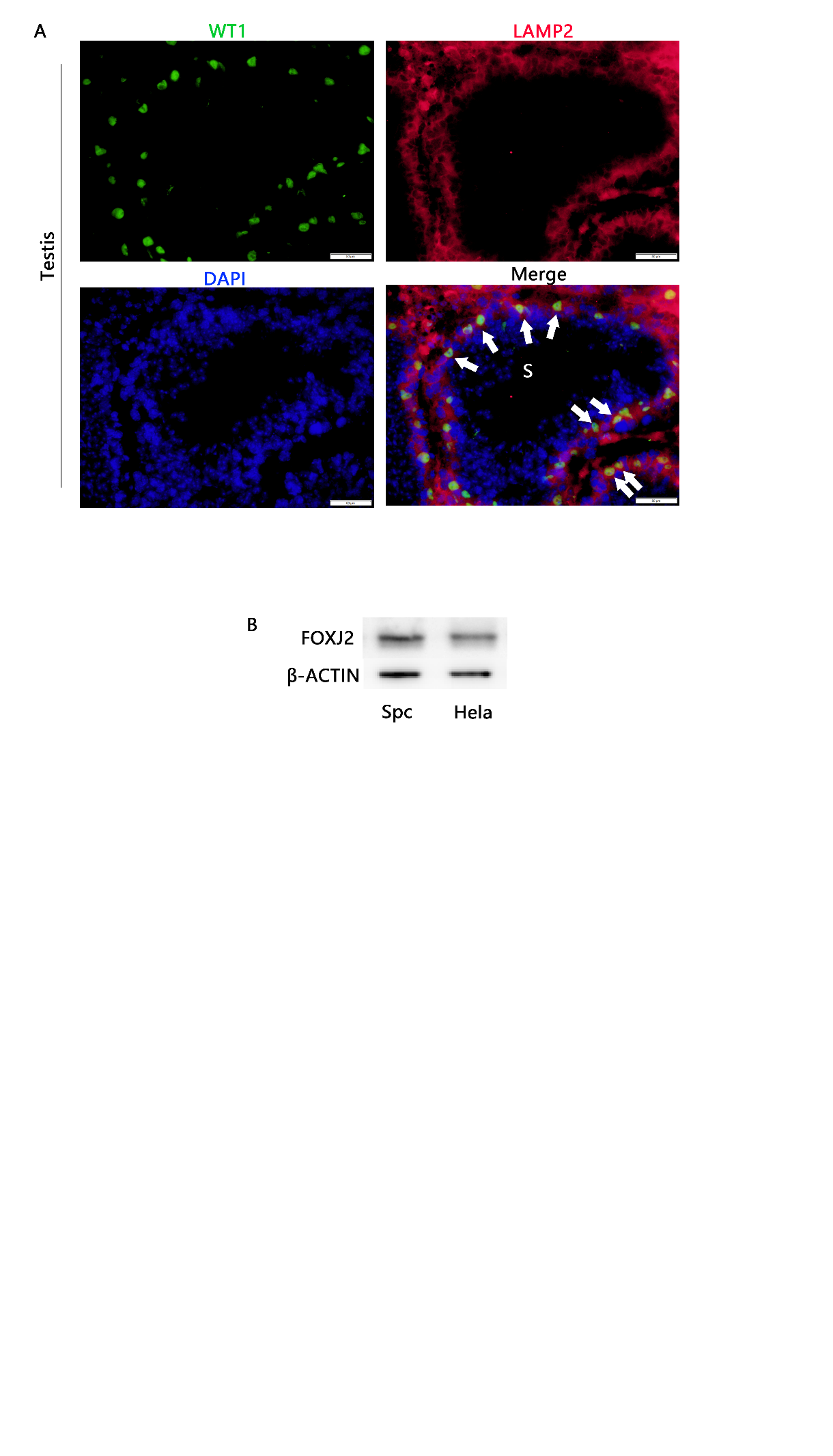


**Supplementary Fig. S4.** LAMP2 locates in Sertoli cells and **Hela cells endogenously express FOXJ2.** (A) Localization of LAMP2 and WT1 (showing Sertoli cells) on testis sections using immunofluorescent staining. Scale bar: 50 μm. White arrows indicate Sertoli cells (S). (B) Western blotting analysis of endogenous FOXJ2 protein expression in spermatocytes and Hela cells. β-Actin was used as the loading control.
